# Cell phone-based online biochemistry and molecular biology medical education curriculum

**DOI:** 10.1080/10872981.2017.1374135

**Published:** 2017-09-13

**Authors:** Junfang Zhang, Zelang Cai, Zhenfu Zhao, Kunmei Ji

**Affiliations:** ^a^ Department of Biochemistry and Molecular Biology, School of Medicine, Shenzhen University, Shenzhen, China; ^b^ School of Medicine, Shenzhen University, Shenzhen, China

We thank Metcalf et al. for the innovative education technology they described in their paper ‘Effectiveness of an online curriculum for medical students on genetics, genetic testing and counseling’,[1] which may serve as our online learning model in education practice. Online education can provide far greater flexibility and convenience than traditional classes.[2,3] There has been a recent growth in the availability of medical curricula for students and the public, as well as a growth in medical continuing education programs, on the internet.[2,3]

Students appreciate innovative educational experiences that can be accessed whenever and wherever is most convenient to the student. They are also calling for more individualized learning formats that take into account the pace of learning, motivation, and learning profile of each student. Meanwhile, use of mobile interactive media, such as smart phones, among students is increasing rapidly owing to the portability and instant accessibility of modern devices.[4] The convenience of mobile internet education enables people to learn anytime and anywhere; the breaking down of the limits of time and space that it represents can be applied to medical education.[5,6]

Biochemistry and molecular biology are important parts of the basic medical curricula and examination subjects covered by the Graduate Candidate Test and National Medical Licensing Examination in China.[7] With the increasing insights into molecular mechanisms of life sciences, the American Society for Biochemistry and Molecular Biology recommends that Biochemistry and Molecular Biology programs develop curricula based on concepts, content, topics, and expected student outcomes, rather than traditional course delineations. However, teaching and learning these curricula are difficult because of the immensity and rapid evolution of the contents. Hence, there is an urgent need for pedagogical methods that enhance learning.[8] To address this need, and taking advantage of the recent progress in internet technology, we developed a mobile phone-based online biochemistry and molecular biology curriculum for medical education in China.

As of May of 2017, there were some 938 million users of WeChat, developed by Tencent Inc., the most popular Chinese social media in China.[9] Apart from providing a free chat application, WeChat’s large-scale network platform hosts a vast amount of user-generated data, including texts, voice calls, videos, and images. Enabled by its immediate communication and source sharing, WeChat offers a convenient medium for delivery of education. We developed and made available a Biochemistry and Molecular Biology WeChat ‘classroom’, which includes online classes, examinations, and recent biomedical progress tracking. The online class function delivers content that is in sync with brick-and-mortar classroom teaching. The examinations are aimed at preparing students for the Graduate Candidate Test and National Medical Licensing Examination. And the recent progress tracking provides students access to information about the newest scientific and technical research advances before such information is published in textbooks. The online class program delivers PowerPoint presentations with voice-over explanations in Mandarin Chinese. Students can review high-resolution images within the PowerPoint presentation on their mobile phones. They can also preview, review, and discuss class materials online easily. The main curricula are consistent with medical major requirements, including clinical medical specialty content, but are also available for students with other majors. The program is gaining student enrolment steadily owing to the curricula being free with open sharing and encompassing interactive communication functionality. Since its introduction on 28 May 2015, 110,000 page views have been accessed. Student feedback indicates that this platform stimulates students’ learning interests and enthusiasm, while also enhancing self-directed learning.

**Figure 1.**
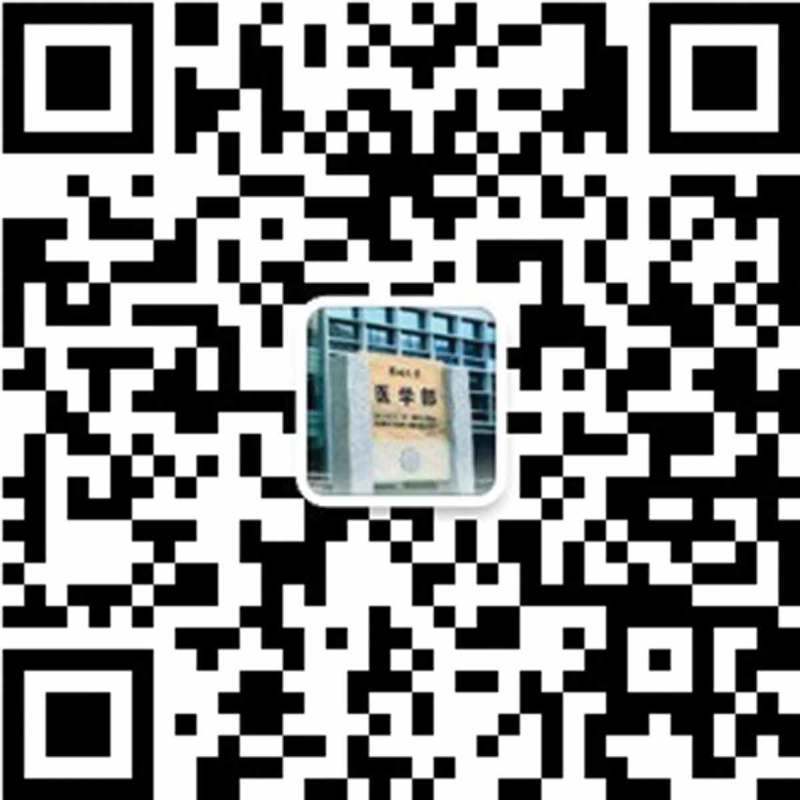
QR code for the free WeChat biochemistry and molecular biology class. To access the online curriculum first go to http://short.weixin.qq.com/ for the Chinese edition or http://www.wechat.com/en/ for the English edition, download and install the WeChat software onto your mobile phone. Second, scan the QR code shown in this figure with WeChat scanner utility.

All teachers and students are welcome to join the Biochemistry and Molecular Biology WeChat class, whose WeChat public code is BioChem_SZU. The quick response (QR) code for accessing the curriculum in shown in Figure 1 and the instructions for enrolling are provided in the Figure 1 legend. We intend to extend the WeChat class enrolment internationally via English-language offerings and to provide access to the class via other social media forums, such as Facebook and Twitter.
